# Integrated lung-diaphragm ultrasound in pediatric weaning assessment: a mini review

**DOI:** 10.3389/fped.2026.1904927

**Published:** 2026-07-23

**Authors:** Yunrui Zhuang, Xingyu Zhu

**Affiliations:** 1 Department of Ultrasound, Tongxiang Maternal and Child Health Hospital, Tongxiang, China; 2 Department of Pediatrics, Tongxiang Maternal and Child Health Hospital, Tongxiang, China

**Keywords:** diaphragm thickening fraction, diaphragm ultrasound, extubation readiness, lung ultrasound, mechanical ventilation, neonates, pediatric critical care, weaning

## Abstract

Weaning from mechanical ventilation remains a major challenge in pediatric critical care. Diaphragm ultrasound has emerged as a promising bedside, radiation-free tool for assessing respiratory muscle function. However, the value of diaphragm thickening fraction (DTF) in neonates and children remains heterogeneous rather than definitive. While several pediatric studies have reported potentially useful DTF thresholds, often around 20%–25%, other cohorts and physiological validation studies have shown weak or inconsistent associations with extubation outcomes or inspiratory effort. These discrepancies likely stem from developmental differences in diaphragm structure and function, high chest wall compliance in children, disease heterogeneity, varying ventilator settings, and technical challenges in obtaining reliable measurements in small patients. Importantly, DTF reflects only one aspect of the respiratory pump and does not evaluate pulmonary factors that increase breathing workload, such as atelectasis, pulmonary edema, consolidation, or poor lung aeration. Lung ultrasound can complement diaphragm assessment by identifying these sources of increased respiratory load during spontaneous breathing trials and after extubation. This mini-review summarizes the current evidence on diaphragm and lung ultrasound for pediatric weaning assessment and proposes an integrated PUMP-LOAD framework: diaphragm ultrasound to evaluate respiratory pump function and lung ultrasound to assess pulmonary load. Although physiologically sound and clinically feasible, this combined approach requires standardized pediatric protocols, age-specific reference values, and prospective multicenter validation before it can reliably guide extubation decisions.

## 1 Introduction

Mechanical ventilation is a life-saving intervention for critically ill neonates and children, yet timely liberation from ventilatory support remains a challenging clinical decision. Premature extubation may lead to respiratory failure, reintubation, prolonged intensive care stay, and increased morbidity, whereas unnecessary prolongation of mechanical ventilation may contribute to ventilator-associated complications, diaphragm deconditioning, and difficulty in subsequent weaning (1–8). Accurate assessment of weaning and extubation readiness is therefore central to pediatric intensive care.

Conventional readiness assessment relies on clinical evaluation, gas exchange, hemodynamic stability, neurological status, secretion burden, airway patency, and spontaneous breathing trial performance (1, 2, 7–10). Indices such as the rapid shallow breathing index have been widely studied, but their role in children remains variable and context-dependent (10–12). These approaches provide important bedside information but do not directly evaluate respiratory muscle function or regional lung aeration.

Point-of-care ultrasound has emerged as a practical tool for respiratory monitoring. Diaphragm ultrasound can assess diaphragm thickness, thickening fraction (DTF), diaphragm excursion (DE), and ventilator-induced diaphragm dysfunction (13–16). DTF has attracted particular interest because it reflects the relative increase in diaphragm thickness during inspiration and is commonly interpreted as a surrogate marker of diaphragm contraction. In adults, systematic reviews and clinical studies have reported favorable diagnostic performance of DTF for predicting weaning outcomes, with commonly proposed thresholds around 30%–36% (17–21).

However, direct extrapolation of adult DTF evidence to neonates and children is problematic. Pediatric patients differ from adults in diaphragm development, chest wall compliance, respiratory rate, respiratory muscle coordination, and disease spectrum (22, 23). Pediatric studies have produced heterogeneous findings: some cohorts found no significant association between DTF and extubation success, whereas others reported potentially useful thresholds such as 17%, 21%, or approximately 20%–26%, depending on measurement side and timing (24–30). Physiological studies have further challenged the assumption that DTF reliably reflects inspiratory effort in children, showing limited relationships between DTF and esophageal pressure or weaning outcome (31, 32).

Successful extubation depends not only on respiratory pump function but also on the pulmonary load imposed after ventilatory support is reduced or removed. Lung derecruitment, atelectasis, interstitial edema, consolidation, pleural effusion, and evolving parenchymal disease may increase the work of breathing even when diaphragmatic thickening appears adequate (33–38).

**Figure 1:**
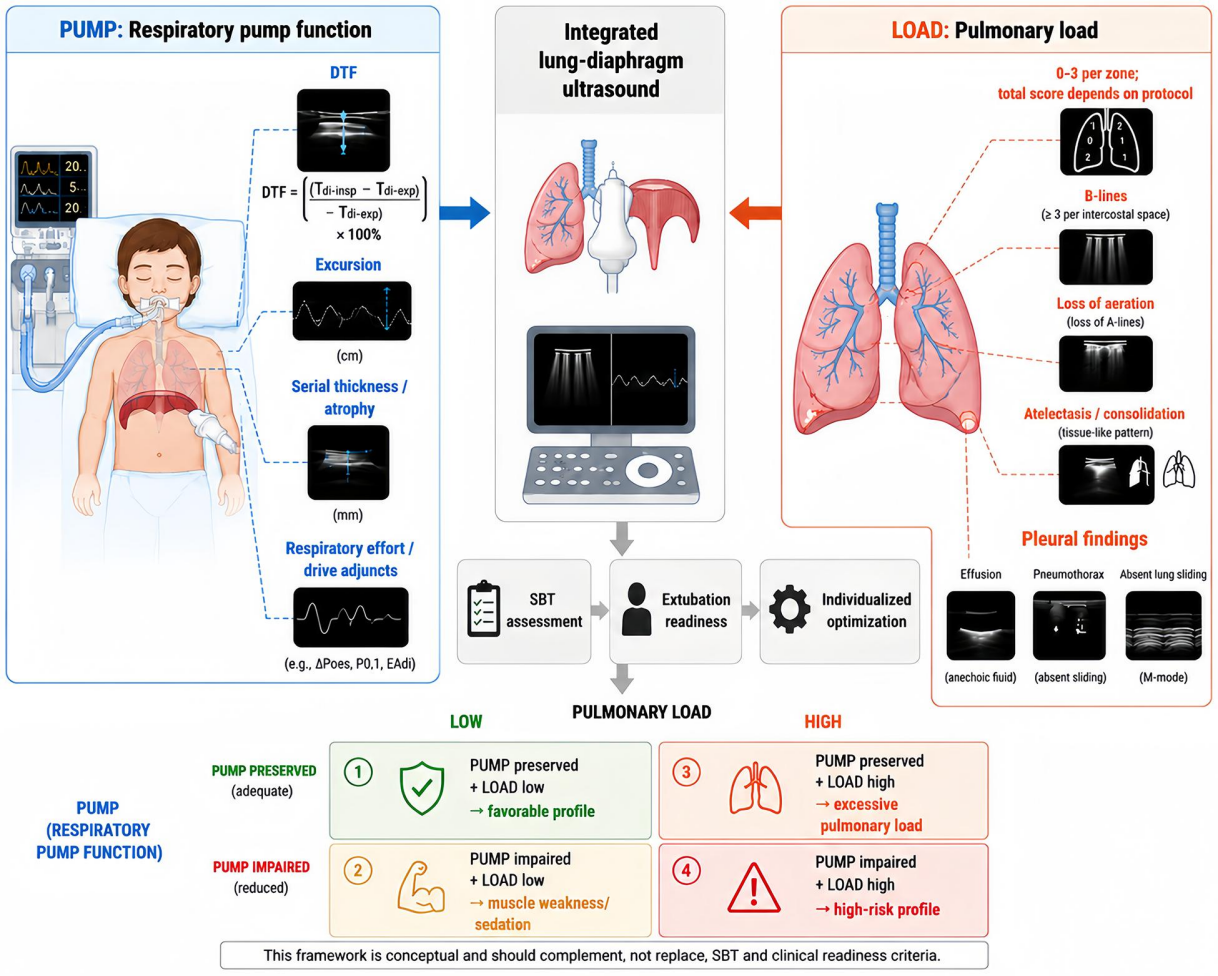
Proposed PUMP-LOAD framework for pediatric weaning assessment. The PUMP component includes patient effort, diaphragm thickening fraction (DTF), diaphragm excursion (DE), and diaphragm thickness or atrophy. The LOAD component includes lung ultrasound score (LUS), B-lines, atelectasis, consolidation, pleural effusion, pneumothorax, and dynamic aeration changes during a spontaneous breathing trial (SBT). Integrated interpretation helps distinguish respiratory pump dysfunction from excessive pulmonary load and supports individualized extubation-readiness assessment. EAdi, electrical activity of the diaphragm; P0.1, airway occlusion pressure measured 100 ms after the onset of inspiration; ΔPoes, change in esophageal pressure.

This mini-review summarizes current evidence on diaphragm and lung ultrasound in pediatric weaning assessment and proposes an integrated PUMP-LOAD approach: diaphragm ultrasound evaluates respiratory pump function, whereas lung ultrasound evaluates pulmonary load. The proposed PUMP-LOAD framework is illustrated in Figure 1.

## 2 Diaphragm ultrasound in pediatric weaning assessment

Diaphragm ultrasound offers a bedside, noninvasive tool for assessing respiratory muscle structure and function during mechanical ventilation. Key parameters include end-expiratory diaphragm thickness, DTF, and DE. Thickness and DTF are typically measured at the zone of apposition with a high-frequency linear probe, whereas excursion is usually evaluated through a subcostal approach using M-mode. DTF is calculated as the percentage change in diaphragm thickness from end-expiration to end-inspiration and serves as an indirect marker of diaphragm contractile activity (13–16).

DTF is appealing because it offers a simple, physiologically plausible measure of respiratory pump activity. Its performance in adults has prompted interest in pediatric use (17–21). The key pediatric studies evaluating diaphragm and lung ultrasound in weaning assessment are summarized in Table 1.

**Table 1:** Evidence summary of pediatric diaphragm and lung ultrasound studies in weaning assessment.

Study	Population and outcome definition	Ultrasound parameters	Main findings	Implication
Duyndam et al. (24)	53 ventilated children, 0–18 years; invasive ventilation >48 h. Extubation success: no reintubation or NIV within 48 h; HFNC was not counted as failure.	Pre-extubation DTF; diaphragm thickness	No significant association between DTF and extubation success; extubation failure occurred in 6 children.	Supports caution against standalone DTF use.
Mistri et al. (25)	55 mechanically ventilated children. Outcomes: extubation outcome and diaphragm changes during ventilation.	Diaphragm thickness; DTF; atrophy rate	Progressive diaphragm atrophy; peri-extubation DTF did not clearly discriminate success from failure.	Atrophy and extubation outcome are related but not interchangeable.
Vadivelu et al. (26)	54 enrolled; 40 planned extubation trials; 9 failures. Extubation failure was mainly reintubation within 48 h.	Serial diaphragm thickness and DTF	Pre- and postextubation DTF were comparable between success and failure groups.	DTF did not reliably predict extubation success.
Xue et al. (27)	50 children with MV >48 h; 39 successes and 11 failures. Success: spontaneous breathing >48 h; failure: SBT failure.	DTF; DE; PImax	DTF ≥21% predicted weaning success; sensitivity 0.82 and specificity 0.81.	Supports potential DTF value in selected settings.
Lee et al. (28)	31 mechanically ventilated children, 1 month–18 years. Extubation failure: reintubation within 48 h.	Daily diaphragm thickness and DTF	Postextubation DTF < 17% was associated with extubation failure.	Useful signal, but the timing limits pre-extubation prediction.
Shah et al. (29)	38 mechanically ventilated pediatric patients during an SBT. Weaning failure: failed SBT or failed extubation requiring reintubation.	DTF during SBT	DTF was lower in failed SBTs; DTF < 25% was associated with SBT failure.	Supports dynamic assessment during an SBT.
Yao et al. (30)	72 children aged 1–3 years; MV >48 h; 56 successes and 16 failures. Success: no ventilation within 48 h.	DE; diaphragm thickness; bilateral and min/max DTF	DE and DTF-derived indices showed moderate prediction; DTF AUCs were 0.71–0.79 with variable thresholds.	Supports DTF utility in a selected age group; overlapping values limit bedside use.
De Meyer et al. (32)	Spontaneously breathing sedated children. Outcome: inspiratory effort under different respiratory conditions.	DTF and esophageal pressure	DTF identified inspiratory activity but did not reliably track graded inspiratory load.	Questions DTF as a graded effort marker.
Chang et al. (31)	Ventilated children. Outcomes: inspiratory effort and ventilator weaning.	DTF and esophageal pressure	DTF did not strongly predict esophageal pressure or weaning outcome.	Provides physiological evidence against standalone DTF use.
Abdel Rahman et al. (37)	106 PICU patients, 1–170 months. Outcome: weaning after an SBT; failure included reintubation within 24–72 h.	DTF; DE; LUS score	DTF, DE, and LUS differed between groups; reported thresholds require cautious interpretation because cutoff direction is inconsistently described across sources.	Supports combined diaphragm and lung ultrasound assessment, but threshold direction should be verified.
Mohsen et al. (42)	Extremely preterm infants; prospective observational extubation study.	LUS; diaphragm ultrasound	LUS accurately predicted extubation outcome; diaphragm measurements were not reliable.	Supports LOAD assessment and integrated interpretation.
Gao et al. (38)	Systematic review and meta-analysis; 11 studies and 828 infants. Success was often defined as extubation for >48–72 h without reintubation; failure included SBT failure or reintubation.	LUS; DTF; DE	LUS sensitivity was 0.88 and specificity 0.81; DTF AUSROC was 0.86.	Suggests that lung aeration assessment is highly relevant in infant weaning.

AUSROC, area under the summary receiver operating characteristic curve; DE, diaphragm excursion; DTF, diaphragm thickening fraction; HFNC, high-flow nasal cannula; LUS, lung ultrasound score; MV, mechanical ventilation; NIV, noninvasive ventilation; PICU, pediatric intensive care unit; PImax, maximum inspiratory pressure; SBT, spontaneous breathing trial.

However, pediatric evidence is more inconsistent. Duyndam et al. found no significant association between pre-extubation DTF and extubation success in mechanically ventilated children (24). Similarly, Mistri et al. and Vadivelu et al. observed diaphragm atrophy and dysfunction during ventilation, yet DTF did not reliably distinguish between extubation success and failure (25, 26). These results support caution against using DTF as a standalone predictor.

Other pediatric studies have reported more positive findings, although with varying thresholds and study designs. Xue et al. showed that DTF ≥21% predicted weaning success (27). Lee et al. found that postextubation DTF < 17% was associated with extubation failure (28). In a pilot study during SBTs, Shah et al. noted lower DTF values in children who failed the trial and identified DTF < 25% as associated with SBT failure (29). Yao et al. reported moderate predictive performance of DE and several DTF-derived indices in mechanically ventilated children aged 1–3 years, with areas under the receiver operating characteristic curve (AUCs) of 0.71–0.79 and thresholds that varied by measurement side and minimum or maximum DTF (30). These studies support potential utility in selected settings, but differences in age, timing, thresholds, and outcome definitions limit broader applicability.

Physiological validation studies have introduced further uncertainty. De Meyer et al. demonstrated that DTF can detect inspiratory activity in sedated children, but increases in inspiratory load led to greater esophageal pressure swings without consistent increases in DTF (32). More recently, Chang et al. reported that DTF did not reliably predict esophageal pressure swings or weaning outcomes in children (31). Because esophageal pressure is a more direct measure of inspiratory effort, these findings question the use of DTF as a standalone surrogate.

Diaphragm ultrasound can also aid longitudinal monitoring. Pediatric studies have documented progressive diaphragm thinning during acute respiratory failure, with links between atrophy, neuromuscular blockade, reduced spontaneous breathing, and prolonged postextubation respiratory support (25, 39–41). Importantly, atrophy and DTF are distinct: a child may show preserved thickening over a brief period yet lack endurance, or exhibit reduced thickness without immediate extubation failure. Diaphragm ultrasound should therefore be interpreted over time and in clinical context, rather than as an isolated threshold-based test.

## 3 Why diaphragm thickening fraction may be context-dependent in children

The heterogeneous performance of DTF in pediatric weaning assessment stems from the complex interplay between developmental physiology, respiratory mechanics, disease heterogeneity, ventilator settings, and technical limitations. In adults, diaphragmatic thickening is generally viewed as a reliable marker of contraction and pressure generation. In neonates and children, however, the link between visible thickening and effective inspiratory effort is less straightforward.

First, the pediatric diaphragm differs markedly from the adult diaphragm in its developmental characteristics. Neonates, particularly preterm infants, exhibit lower fatigue resistance and reduced capacity to sustain force under repeated respiratory loads (22, 23). DTF can capture thickening during individual breaths but does not assess endurance. A child may therefore show adequate thickening during a brief ultrasound assessment or SBT yet fail extubation because the required effort cannot be sustained.

Second, the highly compliant chest wall in infants and young children reduces the efficiency of diaphragmatic contraction. In adults, a stiffer chest wall enables more effective translation of diaphragmatic descent and thickening into negative intrathoracic pressure and tidal volume. In infants, a portion of the generated force is often dissipated through chest wall distortion or paradoxical motion. Consequently, similar DTF values may carry different physiological implications across age groups, making direct application of adult-derived cutoffs inappropriate in children.

Third, successful spontaneous breathing requires more than diaphragm contraction. Intercostal and abdominal muscle function, upper airway patency, respiratory drive, cough effectiveness, secretion clearance, and neurological status all influence extubation outcomes. DTF evaluates only one component and cannot detect upper airway obstruction, inadequate secretion clearance, impaired consciousness, or excessive postextubation respiratory load.

Fourth, pediatric critical illness is highly heterogeneous. Mechanically ventilated children may suffer from acute respiratory failure, congenital heart disease, postoperative respiratory insufficiency, sepsis, neuromuscular disorders, neurological injury, bronchopulmonary dysplasia, or chronic lung disease. These conditions influence respiratory drive, lung and chest wall mechanics, diaphragmatic function, and gas exchange through distinct pathways. A single DTF threshold is therefore unlikely to perform consistently across neonates, infants, school-aged children, and adolescents.

Fifth, DTF measurements are technically challenging in small children. Accurate calculation depends on reliable identification of end-expiratory and end-inspiratory diaphragm thickness, which is difficult given the thin diaphragm, narrow intercostal spaces, high respiratory rates, and irregular breathing patterns. Probe positioning, imaging mode, respiratory phase selection, ventilator triggering, sedation level, patient movement, and operator experience can all introduce significant variability (43, 44).

Ventilator settings further influence results. Pressure support, positive end-expiratory pressure (PEEP), patient-ventilator synchrony, and SBT conditions can substantially alter diaphragm activity. Excessive ventilatory assistance may underestimate a child's true breathing capacity, whereas an overly demanding trial may produce elevated DTF as a sign of distress rather than readiness (29, 45).

Overall, DTF should be interpreted as a contextual physiological signal rather than a binary predictor of extubation success.

## 4 Lung ultrasound as assessment of respiratory load

If diaphragm ultrasound evaluates the respiratory pump, lung ultrasound evaluates the load imposed on that pump. Weaning success requires adequate respiratory muscle activity, sufficient lung aeration, and an acceptable pulmonary workload. A child with preserved diaphragm thickening may still fail extubation if atelectasis, interstitial edema, consolidation, or pleural disease markedly increases the work of breathing.

Lung ultrasound provides a bedside, radiation-free method for evaluating regional lung aeration. The lung ultrasound score (LUS) is the most commonly used semiquantitative approach. Normal aeration with A-lines receives the lowest score, whereas increasing B-lines, coalescent B-lines, and consolidation reflect progressive loss of aeration. A higher LUS indicates a greater burden of poorly aerated or nonaerated lung tissue (33–35).

During weaning, aeration loss may reduce oxygen reserve, increase respiratory drive, and impose a higher load on the diaphragm and accessory respiratory muscles. B-lines during an SBT may reflect interstitial syndrome, lung derecruitment, or increased extravascular lung water. An increase in B-lines may indicate developing pulmonary congestion or aeration loss as support is reduced. Serial LUS assessment before, during, and after the trial may therefore be more informative than a single static scan.

Consolidation and atelectasis are also important causes of weaning difficulty. Dependent atelectasis, postoperative collapse, pneumonia-related consolidation, or mucus plugging may impair oxygenation and increase work of breathing after extubation. Lung ultrasound can identify subpleural consolidation, dynamic or static air bronchograms, pleural line abnormalities, and regional loss of lung sliding. These findings help distinguish a child with preserved pump function but persistent lung disease from one with primary diaphragmatic dysfunction.

Pleural findings may further modify extubation readiness. Pleural effusion, pneumothorax, impaired lung sliding, or marked pleural line abnormalities can affect lung mechanics and gas exchange. Although these findings are not specific to weaning failure, they may identify potentially reversible contributors to post-extubation respiratory distress.

Evidence supporting LUS in weaning assessment is growing, although pediatric validation remains limited. In adults, ultrasound-assessed aeration loss during an SBT predicted postextubation distress (34). In children, Abdel Rahman et al. reported associations between diaphragm and lung ultrasound indices and weaning outcome (37). In extremely preterm infants, Mohsen et al. found that lung ultrasound accurately predicted successful extubation, whereas diaphragm measurements were unreliable (42). A systematic review and meta-analysis in infants also reported favorable diagnostic performance, with LUS showing high sensitivity and specificity (38). These studies suggest that lung aeration is at least as important as diaphragm activity when evaluating readiness for ventilator liberation.

However, LUS should not be interpreted in isolation. Findings are affected by the scanning protocol, number of regions assessed, patient position, timing relative to the SBT, ventilator settings, and underlying disease. B-lines and consolidation are not disease-specific, and lung ultrasound mainly detects abnormalities that reach the pleural surface. LUS should therefore be integrated with oxygenation, ventilation, hemodynamics, secretion burden, airway risk, neurological status, and SBT performance.

## 5 Integrated lung-diaphragm ultrasound: a PUMP-LOAD approach

The limitations of DTF do not diminish the value of diaphragm ultrasound; instead, they indicate that it should be interpreted within a broader physiological framework. Pediatric weaning failure may occur when the respiratory pump is weak, the pulmonary load is excessive, or both conditions coexist. Integrated lung-diaphragm ultrasound can therefore assess the balance between respiratory pump capacity and pulmonary load.

We propose a PUMP-LOAD approach. PUMP refers to respiratory pump function, including patient effort, DTF, DE, diaphragm thickness or atrophy, and performance during an SBT. LOAD refers to pulmonary load, including aeration loss, interstitial syndrome or edema, atelectasis, consolidation, pleural abnormalities, and dynamic changes as ventilatory support is reduced. This framework is intended not to replace clinical judgment or SBTs but to organize ultrasound findings in a physiologically meaningful and clinically practical way.

The PUMP component begins with diaphragm ultrasound. DTF reflects inspiratory thickening during individual breaths, DE reflects craniocaudal diaphragm movement, and serial thickness measurements may reveal ventilator-associated diaphragm atrophy. None of these parameters should be interpreted as an isolated binary threshold; their meaning depends on age, ventilator settings, sedation, respiratory drive, patient-ventilator interaction, and SBT conditions (25, 39–41).

The LOAD component begins with lung ultrasound. LUS, B-lines, consolidation, atelectasis, pleural effusion, and pneumothorax can identify pulmonary abnormalities that increase the work of breathing. A child with worsening B-lines or a high LUS during an SBT may be experiencing increasing pulmonary load even if DTF appears preserved. Management may then need to focus on lung recruitment, airway clearance, fluid balance, positioning, or treatment of infection rather than respiratory muscle performance alone (33–38).

The clinical value of the PUMP-LOAD approach lies in pattern recognition. When both diaphragm ultrasound and lung ultrasound are reassuring, the ultrasound profile supports readiness for extubation if conventional clinical criteria and the spontaneous breathing trial are also favorable. When diaphragm indices are abnormal but lung aeration is acceptable, respiratory muscle weakness, excessive sedation, neuromuscular dysfunction, or ventilator-induced diaphragm dysfunction should be considered. When diaphragm activity appears adequate but lung ultrasound shows significant aeration loss, the primary problem may be excessive pulmonary load rather than pump failure. When both PUMP and LOAD components are unfavorable, the child should be considered at high risk for weaning failure.

This integrated interpretation is especially relevant in pediatrics because DTF alone may be misleading. A high DTF may represent effective diaphragmatic contraction, but it may also reflect increased respiratory drive in response to poor lung aeration. Conversely, a low DTF may indicate diaphragm weakness, but it may also reflect excessive ventilatory assistance, low respiratory drive, sedation, or measurement error. Lung ultrasound helps contextualize whether the diaphragm is working against a favorable or unfavorable pulmonary condition.

A practical workflow may include three time points. Before the SBT, diaphragm thickness, DTF or DE, and baseline LUS can be assessed. During the trial, dynamic changes in DTF, DE, respiratory pattern, B-lines, and regional aeration can be evaluated with clinical signs of distress. After extubation, repeated lung ultrasound may help detect early atelectasis, pulmonary edema, or aeration loss in children with respiratory deterioration. This serial approach emphasizes trends rather than isolated measurements.

## 6 Discussion and future directions

This mini-review underscores the limitations of single-parameter ultrasound for assessing extubation readiness in children. DTF is simple, bedside, radiation-free, and linked to diaphragm contraction, but current pediatric evidence is heterogeneous and does not support its use as a standalone predictor. Variable thresholds, inconsistent clinical correlations, limited agreement with esophageal pressure measurements, and age-related technical challenges indicate that DTF should be viewed as a supportive measure of diaphragm activity rather than a definitive marker of weaning success.

The core clinical takeaway is that successful pediatric weaning requires balancing respiratory pump capacity against pulmonary load. Integrated lung-diaphragm ultrasound may help identify mechanisms of weaning difficulty and guide targeted interventions before extubation. However, it should remain an adjunct to standard readiness criteria, including SBT performance, gas exchange, hemodynamic stability, neurological status, airway patency, secretion burden, cough strength, sedation level, and the child's underlying condition.

Several challenges must be resolved before widespread adoption. Pediatric ultrasound protocols require standardization because studies vary in probe selection, scanning sites, measurement techniques, imaging modes, timing, ventilator settings, and SBT protocols. Age-specific norms are also essential because diaphragm thickness, respiratory mechanics, chest wall compliance, and disease patterns differ across neonates, infants, school-aged children, and adolescents. Structured training is equally important, as small measurement errors can substantially affect interpretation in young patients.

Future research should focus on prospective multicenter validation of integrated ultrasound approaches. Studies should compare DTF alone, LUS alone, DE, and combined PUMP-LOAD models against clinically relevant outcomes, including SBT failure, extubation failure, reintubation within 48–72 h, need for postextubation noninvasive support, mechanical ventilation duration, and PICU length of stay. Predictive models should report discrimination, calibration, and clinical utility because statistically sound models may still lack practical value if poorly calibrated or difficult to apply (46).

Dynamic ultrasound monitoring also warrants investigation. Assessments performed before an SBT may not capture physiological demands during or after extubation. Serial evaluations of DTF, DE, respiratory pattern, LUS, and B-lines may better reflect an evolving pump-load imbalance. Integrating ultrasound with direct measures of respiratory effort, such as esophageal pressure monitoring or airway occlusion maneuvers, would further clarify the relationship between imaging findings and actual inspiratory work in ventilated childre (31, 32, 47, 48).

This review has inherent limitations. As a narrative mini-review rather than a systematic analysis, it lacks formal risk-of-bias assessment and meta-analysis. The pediatric literature remains constrained by small samples, heterogeneous populations, variable outcome definitions, and inconsistent ultrasound methods. Consequently, the PUMP-LOAD framework should currently be regarded as a conceptual and educational tool rather than a validated clinical algorithm.

## 7 Conclusion

Integrated lung-diaphragm ultrasound offers a practical and physiologically sound approach to assessing weaning in children. Diaphragm ultrasound evaluates respiratory pump function through DTF, DE, and serial thickness measurements, whereas lung ultrasound identifies pulmonary load through aeration loss, B-lines, atelectasis, consolidation, and pleural abnormalities. Current evidence, however, indicates that DTF should not be used in isolation to determine extubation readiness in neonates and children.

The proposed PUMP-LOAD framework can help clinicians organize ultrasound findings and differentiate respiratory pump dysfunction from excessive pulmonary load during weaning. Nevertheless, it should serve as an adjunct to SBTs, clinical judgment, and standard readiness criteria rather than as a standalone decision-making tool. Standardized pediatric protocols, age-specific reference values, and multicenter validation are needed before integrated lung-diaphragm ultrasound can be routinely adopted for extubation decisions.
